# Making morbidity multiple: History, legacies, and possibilities for global health

**DOI:** 10.1177/26335565231164973

**Published:** 2023-03-27

**Authors:** Justin Dixon, Emily Mendenhall, Edna N Bosire, Felix Limbani, Rashida A Ferrand, Clare I R Chandler

**Affiliations:** 1 The Health Research Unit Zimbabwe (THRU ZIM), 559150 Biomedical Research and Training Institute, Harare, Zimbabwe; 2 Department of Global Health and Development, London School of Hygiene & Tropical Medicine, London, UK; 3 145743 Edmund A. Walsh School of Foreign Service, Georgetown University, Washington, DC, United States; 4 Faculty of Health Sciences, SAMRC Developmental Pathways for Health Research Unit, 37708 University of the Witwatersrand, Johannesburg, South Africa; 5 Brain and Mind Institute, Aga Khan University, Nairobi, Kenya; 6 560808 Malawi-Liverpool-Wellcome Trust Clinical Research Programme, Blantyre, Malawi; 7 Department of Clinical Research, 4906 London School of Hygiene & Tropical Medicine, London, UK

**Keywords:** Multimorbidity, sub-Saharan Africa, global health, history, social theory

## Abstract

Multimorbidity has been framed as a pressing global health challenge that exposes the limits of systems organised around single diseases. This article seeks to expand and strengthen current thinking around multimorbidity by analysing its construction within the field of global health. We suggest that the significance of multimorbidity lies not only in challenging divisions between disease categories but also in what it reveals about the culture and history of transnational biomedicine. Drawing on social research from sub-Saharan Africa to ground our arguments, we begin by describing the historical processes through which morbidity was made divisible in biomedicine and how the single disease became integral not only to disease control but to the extension of biopolitical power. Multimorbidity, we observe, is hoped to challenge single disease approaches but is assembled from the same problematic, historically-loaded categories that it exposes as breaking down. Next, we highlight the consequences of such classificatory legacies in everyday lives and suggest why frameworks and interventions to integrate care have tended to have limited traction in practice. Finally, we argue that efforts to align priorities and disciplines around a standardised biomedical definition of multimorbidity risks retracing the same steps. We call for transdisciplinary work across the field of global health around a more holistic, reflexive understanding of multimorbidity that foregrounds the culture and history of translocated biomedicine, the intractability of single disease thinking, and its often-adverse consequences in local worlds. We outline key domains within the architecture of global health where transformation is needed, including care delivery, medical training, the organisation of knowledge and expertise, global governance, and financing.

## Introduction

‘Multimorbidity’, defined by the WHO as the cooccurrence of two or more chronic conditions in one individual,^1^ has been framed as among the most significant emerging challenges for health systems globally. The bodies of literature around multimorbidity are weighted towards the challenge it poses in high-income settings, especially among older people for whom multimorbidity is more the norm than the exception. More recently, the focus has broadened to include the challenge multimorbidity poses in countries that share characteristics of lower resources, persisting infectious diseases and dependencies on transnational disease control structures.^2–4^ In such countries, multimorbidity has been argued to pose a unique challenge, characterised by a ‘double burden’ of chronic infectious diseases including HIV and TB and rapidly rising non-communicable diseases, or NCDs (and associated ‘lifestyle’ risks). This is compounded by fragile health systems that remain designed for acute reactive care for single diseases as well as the exacerbating effects of COVID-19.^3^

Research priorities for responding to multimorbidity in a global context and for sub-Saharan African countries specifically have been put together by the UK Academy of Medical Sciences in collaboration with the Academy of Science of South Africa.^2,5^ Priorities include the standardisation of case definitions to enable comparability across studies and disciplines; identification of common disease ‘clusters’ with shared determinants; improvement of the prevention and treatment of multimorbidity; and more broadly, restructuring health systems around the needs of people rather than around diseases.^2,5^ Responding to these priorities requires research that transcends entrenched disciplinary and disease silos, and the inclusion of perspectives from beyond biomedicine, including the social sciences, to respond to the social, political and economic context of multimorbidity.^2,5^ The commitment to standardise definitions and align priorities and disciplines has been echoed and expanded upon by a number of reviews and commentaries.^3,4^ Together, this growing call to action promotes multimorbidity as a new and urgent global health challenge worthy of funding and recognition amidst a field of competing priorities and imperatives.

The commitment to look more holistically at disease interactions is undoubtedly a positive development given the historical predominance of single-disease programming.^6,7^ However, as several commentators have observed, the construct of ‘multimorbidity’ and its apparent rise may say more about tensions within the culture of biomedicine than it does about an objective reality of shifting disease burdens.^8–10^ Moreover, beyond biomedicine, critiques of single disease programming are not new. A wealth of social science scholarship has long highlighted the indivisibility of illness experience and its synergistic interactions with social, ecological, environmental, and political processes – as captured, for instance, within the syndemic framework.^11,12^ Social scientists have also demonstrated why, despite the evident limitations of the single disease model, medicine and global health have nonetheless progressed on a trajectory towards classifying, counting, standardising, financing, and caring for single diseases.^6,7^ Seen in this light, the discursive framing of ‘multimorbidity’ as a relatively new, primarily biomedical health challenge with ancillary need to attend to syndemic interactions that nudge disease clustering is itself worthy of critical, reflexive attention.

In this article, we critically consider discourses around multimorbidity that have been applied to and utilized in global health policy and planning within the context of sub-Saharan Africa. We show how the waters to be navigated can be traced back to the formative architectures of biomedical knowing and forwards to the transnational dynamics that shape systems of care in many sub-Saharan African countries. Highlighting the problematic legacies of single-disease programming in local worlds and the limited successes of interventions to integrate care to date, we argue that current calls to align priorities and disciplines around a standardised biomedical definition of multimorbidity risks retracing the same steps. For attempts to do so presuppose the same problematic categories it exposes as breaking down. We call instead for reflexive, transdisciplinary work across the field of global health around a more holistic, reflexive understanding of ‘multimorbidity’ that centres the culture and history of translocated biomedicine, the intractability of single disease thinking and its often-adverse impacts on lives and livelihoods. We outline key domains within the architecture of global health where transformations are needed, including care delivery systems, medical training, the organisation of knowledge and expertise, global governance, and financing.

## Approach

The analysis presented in this article is based on a non-systematic literature review approach, complemented by insights gained from the author group’s research on multimorbidity and related fields over the past 20 years. Our intention was not to synthesise all that has been published about multimorbidity, but rather to understand how multimorbidity is crystallising as a concept and field of study in the context of global health. As such, rather than adopting a structured framework designed to capture the entire domain of multimorbidity, we followed the approach of similar reviews on multimorbidity^10^ and other health issues^11,13^ that utilised a more open-ended, exploratory approach to the identification and synthesis of literature. This allowed us to trace how multimorbidity is being constructed as an object of science, policy, and care across a variety of bodies of literature in a way that is not facilitated by more structured literature review approaches.

Literature was identified through an iterative process that took as a starting point our own knowledge of the literature base as well as database searches in Ovid Medline, Embase, and Google Scholar. The terms that guided our initial search were "multimorbidity”, “global health”, and “sub-Saharan Africa” (and specific countries from the region), and we included only literature published in English, with no restrictions on date of publication. From initial search returns, we used a purposive and snowballing approach to identify further literature, predominantly by searching bibliographies, citation trackers, and database suggestions for similar articles. As we progressed, we began to see that the way multimorbidity was conceptualised and framed in clinical, public, and global health literature tended to be disconnected from a wealth of literature on multimorbidity and related concepts from across the social sciences. This included insights from anthropology, sociology, history, philosophy, and science and technology studies. Realising that the critical potentials of multimorbidity could be strengthened by putting these bodies of literature into conversation, we proceeded to pull out prominent themes in relevant social science literature, some of which we had contributed to ourselves. The synthesis of our review of current framings of multimorbidity and social science literature forms the basis of the arguments that we present here.

## Making and managing morbidity

Medical philosophers, historians and social theorists have long shown that the idea that different diseases can be treated as discrete, bounded entities is neither objective nor inevitable. Rather, it represents values, interests, and intellectual heritage particular to Euro-Western biomedicine. Influential works by Georges Canguilhem^14^, Michel Foucault^15^, and Nicholas Jewson^16^, among others traced the socio-historical processes through which illness, previously understood to be a unique, indivisible, and subjectively experienced manifestation of an individual’s circumstances, became divisible and objectively discernible within the body. The conditions of possibility for the single disease (and thus for a patient having several of them) have generally been traced back to the birth of ‘modern’ medicine in the 19^th^ century in the new institutions of the clinic and the hospital. During this time, the medical profession organised and sub-divided around different diseases and organ systems. Upon these divisions of medical labour, the ontology of the single disease (beyond its manifestation within the afflicted) was cumulatively solidified by several developments. This included the advent of germ theory, which enabled the attribution of pathological states to simple, universal, externally originating agents.^17^ It also included advancements in statistical techniques that reconfigured ‘the pathological’ from an ideal type or essence to deviation from a healthy norm.^14^ Crucial, finally, was the systematisation of disease classifications in large-scale information infrastructures – notably, the International Classification of Diseases (ICD) – through which such classifications became more transportable, interoperable and taken-for-granted.^18^ The solidification and standardisation of disease categories in turn catalysed a reconceptualization of medicine itself in the 20^th^ century from interpretive ‘art’ to evidence-based ‘science’, wherein clinical practice became increasingly bound to normative (often disease-specific) protocols based on rigorous scientific experiments.^19,20^

The expansion and standardisation of diagnostic categories has led to clinical and public health interventions of important health and social value, leading to longer lifespans to which rising burdens of ‘chronic’ disease and more recently ‘multimorbidity’ have generally been attributed. For as long as this push towards standardisation has occurred, scholars across the health and social sciences have highlighted the adverse consequences of cutting, splicing, and divorcing the body from history, society, and social relations. This is not least because such processes frequently serve financial and political imperatives in which the needs of patients become secondary.^21^ The disjuncture between patient needs and other interests has become especially stark as categories of ‘the pathological’ have been increasingly projected into the future through statistical configurations of risk.^22^ Anthropologists, in particular, have highlighted that the ability to index both current and anticipated health conditions to standardised ICD classifications has enabled a vast medical industrial complex and rapid expansion of pharmaceutical markets. This has been especially evident in the industrial centres of the global north, but is increasingly the case globally.^21^ It is notable against this backdrop that a recent review of narratives and responses to multimorbidity has shown that its apparent rise may be as much an artefact of the expansion of disease and risk categories within biomedicine as much as any ‘true’ rise in disease burdens.^10^ This not only leads to habitual overdiagnosis and ‘polypharmacy’, presenting challenges for patients and providers at the clinical level.^10,23^ The new configurations of risk and responsibility enabled by these categories also makes disease classification pivotal to the extension of modern political power at the population level. Referred to as ‘biopolitics’, this entails the naming and defining of people and populations as entities for management by the state.^15,17,24^

The biopolitics of disease classification have taken on a distinct and problematic trajectory in Africa. Fanon^25^ and others implicated biomedicine in the expansion of colonial rule, showing how medical authorities concerned with ‘native health’ overwhelmingly prioritised maternal and child health and diseases classified as ‘infectious’ in the interests of sustaining a productive labour force.^7^ These tensions were extended into the early post-colonial era. The WHO, UNICEF, and invested states debated the virtue of quick technical fixes versus universal, comprehensive primary healthcare, with commitments to the latter concretised with the Declaration of Alma Ata.^7^ Commitments to elevating national health systems were quickly undermined, however,^26^ by the widespread imposition of neoliberal macroeconomic policy (i.e. policy favouring free-market capitalism and reducing government spending) in the 1980s and 1990s. These policies systematically defunded public sectors and shrunk them in favour of privatisation.^6,7^ A well-rehearsed narrative in the interdisciplinary field of critical global health charts a rupture in the biopolitical order. This is characterised by a transfer of sovereignty in matters of health from national governments and the WHO to the new financial powerhouses of the emerging field of ‘global health’ including northern governments, non-governmental organisations (NGOs), and philanthropies. Early on, these transfers of financial contributions were attributed to hot-button issues like population control and eventually HIV and AIDS. However, in contrast to the visions of Alma Ata, these institutions favoured investment in narrow technological interventions to address priority infectious diseases with demonstrable return on investment. Countervailing voices in both applied and academic spheres have continued to advocate for comprehensive primary care and broad-based health system strengthening. However, such financial and technological imperatives led to a proliferation of ‘vertically’-organised surveillance sites, population trials and clinics across Africa’s health systems. In such settings, diseases prioritized in global agenda setting, notably HIV, TB, and malaria have been extremely well funded, and some of these resources have expanded community-based care infrastructures.^27^ However, these global agendas have largely left other areas of health grossly underfunded and specialised.

Discourses of ‘epidemiological transition’ have played an important role in recent years in increasing the visibility of and attention to NCDs, including mental illness. Elevation of NCDs has particularly developed through statistical advances including the measure of the disability-adjusted life year (DALY), the unit of the influential Global Burden of Disease Study.^28^ However, such discourses of ‘transition’ remain problematic. Vaughan et al. argue that the appearance of transition is at least partially because chronic disease was for so long simply not counted due in part to racialised assumptions about Africans being immune to ‘diseases of civilisation’.^28^ While NCDs are now increasingly counted and accounted for within contemporary surveillance architectures, similar imaginaries of difference persist through a narrow focus on particular NCDs that can be connected to ‘modifiable risk factors’ (i.e., smoking, substance abuse, sedentism, and unhealthy eating). This ‘lifestyle drift’, defined within the contours of victim-blaming, has invited imaginings in the African context of ‘maladaptation’ to modern living. Such imaginings contributed to shifting responsibility onto individuals and communities, disincentivising meaningful investment in these areas and further drawing attention from other conditions that do not fit within these reductionist disease categories.^28,29^ Against this backdrop, the uniqueness of multimorbidity in low-resource settings has been characterised by the ‘double burden’ of persisting infectious diseases interacting with rising NCDs. This challenges any straightforward linear transition from ‘communicable’ to ‘non-communicable’ disease and points towards their co-existence within cross-cutting disease ‘clusters’.^28^ However, the question remains as to whether efforts to integrate care under this umbrella of cross-cutting disease clusters will be sufficient to meaningfully unsettle the prevailing biopolitical order. To emphasise the nature and intractability of the challenge that multimorbidity interventions need to be responsive to, we next show how these classificatory legacies continue to shape the lived experiences of patients, providers, and health authorities in particular settings in sub-Saharan Africa. Such material realities must, inevitably, form the starting point for meaningful steps towards a genuinely transformative response.

## Classificatory legacies

Box 1a presents the story of Grace, a 50-year-old woman living in Kibera informal settlement in Nairobi, Kenya. The fact that Grace could have such different experiences of receiving care for HIV compared to diabetes and hypertension – and indeed, that these diseases are parsed out from her at all – is intimately connected to the classificatory biopolitics described above. Grace might experience her illness holistically as the manifestation of poverty, stress, toxins, infection, and other external factors in her socio-material environment – terms for which include ‘syndemic suffering’^30^ and ‘local biology’.^17^ But from a biomedical perspective she is defined by her diseases. Such reductionism, cut and splicing not only fail to capture the nature of her health and social problems but also shape and exacerbate her situation.

Box 1. Disease-centred versus person-centred care in Kenya.

**Table:** 

1a. Patient experience of the current disease-centred care system in Kenya*	1b. Counterfactual scenario of person-centred care in Kenya
Grace is a 50-year-old woman living in Kibera informal settlement in Nairobi, Kenya. Following the passing of her husband, she is the main provider for her family including her three children, which she does by selling food stuffs in her neighbourhood. She suffers from HIV, diabetes, and hypertension. In contrast to HIV, which she finds relatively easy to manage and for which her care and medicines are free, she is less able to control her diabetes and hypertension. This is especially so given that she must pay out-of-pocket for many of the costs associated with these diseases. The quality of care, she feels, is lower, the demands on her more, and medicines, particularly insulin, are expensive. She complains of body aches and other health-related difficulties that prevent her from earning money to support her family. Recently, she was admitted to hospital for two weeks, but asked the doctor to discharge her because the care was not good, and she was not improving. She is tired, fatigued and stressed most of the time.	In a person-centred health system, Grace is treated as more than a sum of her individual diseases. Medicines and care for hypertension and diabetes are free and, alongside HIV, these conditions are monitored through a single appointment at the primary care centre by the same nurse, in close collaboration with the community health worker. Hospital visits for Grace are few and far between, and when she has needed to go the hospital, she has been seen quickly and her experience has been positive. Her diabetes and hypertension are under control, and she has since had more energy and experiences less pain. The primary care centre is cognisant of Grace’s role as the main provider for her family and has coordinated with the department of social development to ensure that she receives financial and nutritional support. She is also financially better off not having to pay out-of-pocket for medicines, transport, and expensive specialist hospital care. She has more time to work, more time for family and community life, and overall feels positive.

*Case adapted from Bosire et al.^30^

Anthropologists have long grounded critiques of ‘vertical’ programming in immersive ethnographic studies of how people like Grace navigate the resulting fragmented, donor-dependent healthcare systems in practice. Several ethnographic and qualitative studies including in Kenya,^31^ Malawi^32^ and South Africa^33^ have shown the challenges and workload that is placed on patients to manage unevenly prioritised conditions. This includes: making sense of multiple diagnoses framed through medical terminology; navigating multiple providers (formal and informal, biomedical and traditional); accessing family and community support; paying significant out-of-pocket costs for medicines and private care; and managing lifestyle changes and multiple medicine prescriptions. Importantly, many of the challenges of multimorbidity in higher-income settings have been attributed to overdiagnosis and polypharmacy.^10,23^ By contrast, Chikumbu et al. have highlighted that a fundamental driver of the ‘treatment burden’ of multimorbidity in Malawi is the sheer *lack* of access to medicines and care.^32^ Based on fieldwork in West Africa, Vinh-Kim Nguyen developed the concept of ‘therapeutic citizenship’ to capture the extent to which access to medicines, care, and resources hinges thinly on having been diagnosed with HIV, or another disease prioritised by northern research and programme funders. This can provide validation, meaning, and hope for many living with diagnosed conditions. Equally, it can place considerable responsibility on individuals to align their lives, lifestyles, and views of self to fit the requirements of medicine regimens, while excluding and silencing people, problems and understandings of ill health that do not fit with donor priorities and diagnostic criteria.^34,35^

While much qualitative research has focused on patients, an increasing body of ethnographic research in sub-Saharan Africa has shown that the detrimental effects of ‘vertical’, single-disease programming are felt ‘all the way up’ health systems.^36,37^ It presents a challenge for the nurses and community health workers who provide the majority of care at primary level in sub-Saharan Africa. These frontline actors are trained to adhere to disease-specific clinical practice guidelines that have limited applicability to patients with multimorbidity^38^ and whose attendant paperwork and accountability draw time and attention away from the complexity of patient needs.^39–44^ Understandably, healthcare workers tend to gravitate towards NGO facilities – which tend to be specialised and disease-specific – rather than high-pressure, underpaid public facilities where they are most needed.^27^ Focusing on specialised care also limits the options and opportunities for scientists. Their careers are generally pulled towards external disease-specific agendas and, despite the language of ‘equitable partnership’ in transnational research collaborations, they tend to perform the ‘grunt work’ of implementing protocols rather than meaningfully engaging in the design and direction of research.^26,45^ Importantly, it also undermines the authority of local health planners and policymakers, who are increasingly beholden to US-based or -trained health financing and policy advisors promoting market-based health systems or private-public partnerships.^46–48^ While many work towards local needs,^37^ global health agendas are so overwhelmingly stacked towards neoliberal interests that it is almost impossible for even those who want to advocate for more integrated care to have a meaningful say in how, where, and by whom care is delivered.

Against this backdrop, a number of frameworks have been developed to bring about more integrated, person-centred care for multimorbidity, including in low-resource settings.^49,50^ However, efforts to actualise such care have had limited success. Robust trials on patient-centred care models for multimorbidity in high-income contexts, for instance, have notably failed to make significant improvements in patient outcomes.^51,52^ Lynch et al. found that an increasing focus on multimorbidity in the UK’s health sector has paradoxically resulted in greater complexity and fragmentation of services. It also perpetuated a disease-centred biomedical gaze focused on bodies rather on social relations and realities of patients.^8^ Interventions explicitly designed to respond to multimorbidity in sub-Saharan Africa, currently much fewer in number, have similarly experienced challenges. In an ethnographic study of South Africa’s Integrated Chronic Disease Management programme, Bosire et al. showed that health workers perceived the programme’s aim to provide person-centred care as important, which they interpreted in relation to the principle of Batho Pele (roughly translated from Sotho as People First).^44^ However, they were left to manage the gap between programme goals to ‘activate’ patients to take responsibility for their health and the reality that, in practice, the programme had not fundamentally addressed the structural conditions that would enable such changes to happen. This included staff shortages, fragmented services and clinical practice guidelines, persisting poverty and language barriers.^44^ These studies demonstrate how neoliberal logics sustain siloed clinical interventions that may be further exacerbating the fragmentation and disease-centricity of management approaches.

## Reframing multimorbidity in global health

A focus on multimorbidity is hoped to challenge siloed, verticalised disease programming and enable more holistic, upstream and ‘patient centred’ approaches to medicine and global health. However, the history of disease classification and its legacies in African healthcare systems suggest that constructing multimorbidity from the same disease categories it is hoped to challenge may be counterproductive. For it risks reproducing entrenched classificatory (in)visibilities and neoliberal renderings of self, health, and responsibility. Similarly concerned that foregrounding disease ‘clusters’ and ‘risk factors’ could perpetuate the status quo, Blarikom et al. have recently proposed a more holistic, reflexive reconceptualization of multimorbidity. They consider multimorbidity not as a compound disease category, but as an “experience that manifests through the discrepancy between medical policy and life-as-lived, brought to the fore by people’s attempts to bridge fissured care systems”.^10^ Accordingly, they carefully draw out the benefits of a social navigation approach that centres social research in documenting the lived experience of contending with multimorbidity in practice.^10^ Based on our analysis of the sub-Saharan African context, we would emphasise that such a wide-angle lens on multimorbidity has the potential to shine light not only on the suffering of patients and the challenges they face navigating vertical, northern-driven health systems. It also reveals the tensions, challenges, and epistemic injustices such programming creates across the field of global health, from patients, to providers, to scientists, to health planners and policymakers. In what follows, we sketch some of the implications of such a reframing for shifts in the architecture of global health that could contribute to reducing tensions between policy and life-as-lived in particular settings. We start closest to the ground with the organisation of care, before moving our focus further upstream.

### Actualising care for whole persons

Among the challenges of actualising aspirations for patient-centred care for multimorbidity is that interventions’ engagement with ‘the social’ often extends only as far as behaviour, culture, and lifestyle factors. In many cases, this results in a profound undermining of individual-level efforts by victim-blaming and shaming patients from engaging in self-care.^53^ Thus, interventions fall short of challenging the structural conditions that lead to multimorbidity and increase treatment burdens.^44^ While such structural factors are often considered to be beyond the purview of biomedicine, there is precedent for such broader-based approaches. One example is the Rwandan government’s work with Partners in Health and the Clinton Foundation, a collaboration which designed integrated NCD care for people living with HIV and AIDS in a rural, decentralised healthcare system.^54^ A central component of this programme was recognising that HIV is a social disease that needs to be addressed not through behavioural and lifestyle interventions but by targeting its roots in social and economic marginalisation. Another example is AMPATH, a partnership between Kenyan and US medical schools, the Kenyan government, and other organisations that integrated medical care with nutrition and family support, education, counselling, insurance, jobs, and financial independence.^55^ Examples such as these remain few, have generally been financially well-supported, and are thus not representative of the care received outside these programme contexts. But they show that models that collapse the distinction between health and social problems can be achievable in low-resource settings.

Programmes grounded in social theory have been especially cognisant to build structural and systemic factors into their models of change. Partners in Health, for instance, have long been influenced by founder Paul Farmer’s work on structural violence.^56^ Another recent care model for actualising patient-centred care for multimorbidity is that of syndemic care.^57^ Undergirded by the principles of syndemic theory, syndemic care requires recognising how social problems cluster with and affect medical problems, and that co-occurring problems can present differently than singular problems, in highly context-dependent ways.^57^ In some cases, syndemic care requires the integration of legal protection for patients who do not feel safe due to policies around policies for migrants, refugees, or asylees.^58,59^ Syndemic care views patients as one unit as opposed to a composite of discrete diseases. Treating patients as one unit entails coordinated medical visits provided by a single medical centre and caregiver rather than multiple disease-specific centres. It also means decentralising care through task-sharing initiatives such that services historically only manageable by doctors can be provided by nurses and community health workers near patients’ homes. Rather than disease specific guidelines, health workers are trained in holistic health models that integrate diagnosis and treatment of physical, mental, and social problems. Community health workers, in the syndemic care model, actively screen for conditions that commonly cluster together (e.g., diabetes and hypertension or depression) and other risk factors that are most common in that particular setting. This requires reprioritisation to decentre high-profile diseases (e.g., HIV, TB, and malaria) to include NCDs and other infections, as well as social conditions including nutrition, substance above, overcrowding, and weak social networks. Once priority conditions are identified (or negated) by general screening procedures, an integrated treatment plan is established at primary care level with recognition of the porosity between health and social care. Box 1a describes how care for persons might be operationalised to manage Grace’s illnesses within the context of the Kenyan health system.

The implementation of models to care for whole persons rather than for diseases is key to bridging the discrepancy between medical policy and life-as-lived. However, building such models into national and transnational systems of care in ways that are tailored to particular epidemiological, socio-economic, and health system contexts, in turn compels a plethora of further transformations within the architecture of medicine and global health. This includes medical training, the organisation of knowledge and expertise, global governance, and financing.

### Reshaping medical training

Learning to care for whole persons needs to start from medical training contexts, which is currently more emphasised in nursing than in medical schools. An established canon of sociological and anthropological literature in high-income settings has examined the formal and hidden curricula through which students learn to ‘see’ and ‘be’ like a physician.^60–62^ Perhaps caricatured and certainly not universal, this literature describes the transmission of a moral order characterised by increasing detachment. Students learn to disassociate from situated, suffering individuals to see only the somatic body, body parts and disease entities as laid out in anatomical curricula.^60,63^ Currently physicians learn their place ‘above’ the supporting cast of nurses and allied professions. Hospital-based specialities in this imagining sit the apex of medical and academic achievement over and above more generalist specialties and public health.^64,65^ Studies of medical training in sub-Saharan Africa add to this picture with additional complications of the legacies of translocated training and navigation of limited resources in their medical socialisation. Students are often taught using outdated Euro-Western textbooks.^63^ Yet, they are forced to sink or swim in under-resourced health systems in which doctors are stretched extremely thinly and the majority of care is task shifted (usually through disease-specific clinical practice guidelines) onto nurses and community health workers.^27^ Studies in physician and nursing training contexts suggest that students may experience empowerment, opportunity and upward mobility – for some, opening doors to specialist training and employment overseas. But they must also navigate status ambiguity, resource-limitations, disenfranchisement, and frustrations about being unable to do more.^63,66–69^ In an ethnographic study of medical education in Malawi, Claire Wendland showed that in contrast to the trajectory towards objectification and disengagement found in high-income settings, Malawian students became more compassionate, empathetic and politically active through their training.^63^ Whilst such studies provide rich insights into the creative adaptation of biomedical knowledge in training contexts, there remain few accounts of medical education compared to clinical practice and research.

An insight brought to the fore by discussions around multimorbidity is the need for a shift in the training of medical professionals away from the predominant ‘specialism’ perspective towards greater generalism in medical practice, to transform how illness is understood and care is delivered.^2,70^ Fundamental to the values and provision of primary care, generalism involves the ability to manage undifferentiated illness and a wide range of conditions, to see the person as a whole in the context of their family and wider environment, to take on responsibility for continuity of care, and to coordinate care within and between health and social care institutions.^71,72^ Conceived as such, the elevation of generalism aligns with the increasing emphasis in medical training on cultural and more recently structural competency.^73,74^ Greater emphasis on generalism in medical training is also a necessary condition for actualising syndemic care.^57^ Although a generalist gaze is already compelled by the material realities of health work in resource-limited settings (even among hospital-based specialists), there is need for greater support for the development of such skills across the spectrum of the health professions. This includes strengthening and increasing collaboration between training programmes for physicians, nurses, and community health workers. It also entails the capacitation of multi- or trans-disciplinary teams to provide preventative and curative care at community level.^71,72^ South Africa, already ahead of the curve, has trained ward-based outreach teams of physicians, nurses, and community health workers,^75^ strengthened generalist training within the HIV platform through programmes such as SWITCH (Strengthening the Workforce to Improve Treatment and Care of HIV) and sought to align care roles and responsibilities through its Integrated Chronic Disease Management programme.^76^

In high-income settings, there has been some pushback against attempts to move towards greater generalism in medicine for reducing specialist opportunities. This happened, for instance, among medical students in the UK in response to its Shape of Training programme.^77^ Accounts of medical socialisation in sub-Saharan Africa suggest that there may be greater support and political will among students for the strengthening of generalist training given the resource-constrained settings in which they must work. But with relatively few studies of emerging medical education paradigms in Africa^78^, of how students perceive the relative importance of specialism and generalism and more broadly the place of the medical profession in society,^63^ the possibilities for reform in medical education is difficult to gauge. There is clear need to ensure that generalism becomes inspiring to future generations of healthcare professionals, its complexity embraced and its importance as a field of expertise recognised.^71^ While undoubtedly an interdisciplinary undertaking, social scientists, with an acute sensibility to both formal and hidden curricula as well as the political economy of knowledge that has come to favour specialist trajectories, will be an important part of efforts to understand and advance this aspect of medical education in the coming years.

### Dissembling knowledge siloes and hierarchies

A further issue that shapes the contours of possibility for shifts in training curricula and the organisation of care is way biomedical knowledge and expertise has been progressively built up around single-disease and organ-system classifications since the 19^th^ century. In biomedicine, as in science more generally, knowledge has tended to be produced within particular ‘epistemic cultures’ with their own objects, histories, styles of reasoning, validated forms of expertise and ways of generating evidence.^79^ Such cultures have come to be typified at the clinical level by the ‘-ologies’ (neurology, cardiology etc.). These have evolved as parallel structures with considerable autonomy, albeit with ever greater complexity, sub-specialisation and jurisdictional contestation following technological advancement, longer life spans, market logics, and globalisation.^80–82^ At the level of public and global health, meanwhile, epistemic cultures also manifest through the transnational communities that have formed around particular high-profile disease classifications. Such communities have their own branding, identities, languages, and frameworks and compete fiercely for visibility and funding.^83^ Their global reach is enabled by vast assemblages of governance structures, funding streams, conceptual frameworks, surveillance platforms, research centres, specialist clinics, clinical practice guidelines and more. Often running parallel to national health systems and infrastructure, these assemblages converge and connect to produce, legitimise, and sustain the ontology and visibility of a particular disease.

The recent rise of interest in multimorbidity seems to be functioning as a beacon bringing together researchers and practitioners from across the architecture of medicine and global health who are struggling with the status quo. Much of the current activity around multimorbidity – attempts to harmonize definitions and priorities,^2,3,5^ foster interdisciplinary collaboration,^2,5^ integrate research and routine health information platforms,^84^ to devise more person-centred health data and care models,^85^ and facilitate more equitable and inclusive partnerships and agendas^5^ – can be read as attempts to build conceptual and infrastructural bridges across epistemic cultures and to begin to reverse knowledge flows. This is a welcome development and an important step towards decentring the single disease. However, a problem that several commentators have noted is that the ability to mount a coordinated response is assumed to depend on first settling on a common definition and core set of conditions that make up multimorbidity.^10^ Even if consensus on a definition specific to multimorbidity in low-resource settings is reached, our historical analysis of the biopolitics of disease classification suggests that organising disciplines around a such a definition will not fundamentally unsettle the problem of defining and prioritising people by their diseases. By the same token, such configurations risk continuing to reduce ‘the social’ to behaviour, culture, and modifiable lifestyle factors. This in turn may continue to side-line disciplines, perspectives, and forms of evidence that have most potential to bring medicine and global health back into closer alignment with the complex lived realities of patients and providers.

Reconceptualising multimorbidity more holistically as a discrepancy between life-as-lived and medical policy,^10^ by contrast, compels a decentring of diagnostic taxonomy and the elevation of perspectives that are able to bring this lived discrepancy to the foreground. This includes perhaps most notably perspectives from the social sciences, whose multifaceted contributions to understanding, reframing and suggesting ways out of the current predicament we have already described in detail here. But it also includes a wealth of public health scholarship since the Whitehall studies that has highlighted the synergistic interactions between embodied illness experience and external circumstances.^11,12^ It further includes work within the primary care sciences that has compellingly adapted the principles of complexity theory to health and to multimorbidity specifically.^9,86^ This is not to mention the vast local knowledge, expertise, and experience that does not make into peer-reviewed journals and is often bypassed in the design of interventions. Folding complexity and context-sensitivity into our framing of multimorbidity presents an opportunity to bring together disciplines, fields, and perspectives both within and beyond biomedicine in novel and potentially unexpected configurations to articulate and respond to people’s needs. Such configurations may, indeed, vary considerably to respond to particular histories, health system contexts, and conceptions of self, illness, and health. However, such context-near health work is precisely what the challenge of multimorbidity arguably compels. Indeed , it offers a more promising route towards realizing aspirations for ‘person-centred’ (or perhaps better, ‘syndemic’) care than harmonizing disciplines and priorities around a standard biomedical definition.

### Shifts in global governance and funding

Transformation in how people with multimorbidity are perceived and cared for will ultimately only be possible with shifts in the governance structures of medicine and global health. As described above, fragmented health systems in sub-Saharan Africa can be traced back through a long and problematic history of Euro-Western influence in health. This was first through direct colonial rule and, more recently, through the technological and financial imperatives of global health that have undermined the growth or development of health systems. Many of the hopes pinned to multimorbidity to bring about more holistic, ‘person-centred’ care resonate with long-standing socialist health agendas for broad-based health system strengthening and comprehensive primary care.^2,5^ Perhaps precisely because multimorbidity is formulated in disease-centric language, it does seem to possess a degree of ‘charisma’ that has often been lacking from calls for more integrative approaches to health. As a result, it has been relatively successful to date in garnering funding and political will. The COVID-19 pandemic has further underscored the importance of attending to disease interactions. For better or worse, the pandemic has demonstrated that addressing ‘underlying conditions’ that have historically been neglected might just be in the interests of (trans)national health security.^87,88^ However, the construction of multimorbidity in policy, as in science and medicine, as a matter of diseases rather than people may not be enough to disrupt neoliberal regimes of funding, counting and accounting. It is these that continue to perpetuate ‘verticalized’ technological approaches, disempower national governments and place responsibility on individuals and communities to secure their own health. Our analysis and those of numerous others demonstrate that the historical trajectories that came to favour such approaches were neither natural nor inevitable, nor are their legacies impossible to undo. Sitting at a critical moment of fracture in global governance and funding brought about by COVID-19, now is an unprecedented moment to instil in funding institutions, transnational health organisations and national governments the health, social, and economic value of a shift back towards more holistic, comprehensive approaches to care. Mobilising around a multimorbidity concept that foregrounds the history and multifaceted violence that siloed, ‘vertical’ disease programming continues to inflict in local worlds and up through low-resource health systems could be powerfully leveraged towards this end.

## Conclusion

In this article, we have examined the construction of multimorbidity within global health, with a focus on sub-Saharan Africa. Drawing on a wealth of social theory and research, our aim has been to expand the multimorbidity conversation to include critical, reflexive consideration of the histories and legacies that sustain disease-centred health systems and that continue to sit within current framings of multimorbidity. To avoid retracing the same steps, we have proposed a shift in focus away from seeking to align priorities and disciplines around a standardised biomedical definition of multimorbidity. Instead, we suggest mobilising around a more holistic, reflexive understanding of multimorbidity as experienced discrepancy between medical policy and life-as-lived,^10^ a discrepancy which manifests differently across contexts, settings, and histories. Such a reconceptualisation points towards transformations in the architecture of medicine and global health that would be needed to realise long-standing aspirations for more holistic, person-centred care. This includes shifts in the organisation of care delivery, the shape of medical training, in the organisation of knowledge and expertise, and in global governance and funding (summarised in Table 1). Reflexive, transdisciplinary work across these different domains in different settings offers hope for reducing this discrepancy such that the idea of ‘multi’ ‘morbidity’ may begin to lose its purchase on patient realities. Perhaps it may cease to make much sense at all.

**Table 1.** Transformations in the architecture of global health needed to respond to multimorbidity.

Domain	Current systems	Future systems
Concepts of health and disease	• Predominance of the single disease model • Multimorbidity defined within the contours of the single disease model as two or more chronic conditions	• Recognition of the indivisibility of illness experience • Holistic, reflexive concept of multimorbidity as discrepancy between policy and life-as-lived^10^
Care delivery systems	• Patients defined by their diseases • Care fragmented, centralised, and costly for patients and health systems • Curative, pharmaceutical interventions for priority infectious diseases • Behavioural, cultural, and lifestyle risk factors foregrounded rather than patient social circumstances and needs	• Patients seen as a whole in their wider context • Care integrated and provided at primary and community levels • Structural vulnerabilities foregrounded and addressed, collapsing the distinction between health and social care
Medical Training	• Anatomical curricula organised around diseases and organ systems • Specialist trajectories favoured and aspired towards • Hierarchical ordering of cadres of healthcare professionals	• Learning to care for whole persons • Elevation of generalism as a field of expertise • Flat relationship between members of multi- or trans-disciplinary teams
Knowledge and expertise	• Produced and enclaved within siloes • Global, vertically-configured knowledge infrastructures • Disconnected from local expertise, knowledge, and infrastructure	• Interdisciplinary and intersectoral working • Knowledge and expertise organised to fit needs and realities in particular contexts • Elevation of the social sciences and other disciplines foregrounding patient realities
Governance and funding	• Priority setting and funding driven by technological, financial and economic imperatives in the global north • Funding vertically organised around high-priority diseases • Bypassing of the sovereignty of national governments	• Health priorities driven by national governments and local actors, supported where needed by international partners • Funding for broad-based health system strengthening and comprehensive care rather than diseases
